# Aggressive Transition between Alternative Male Social Tactics in a Long-Lived Australian Dragon (*Physignathus lesueurii*) Living at High Density

**DOI:** 10.1371/journal.pone.0041819

**Published:** 2012-08-08

**Authors:** Troy A. Baird, Teresa D. Baird, Richard Shine

**Affiliations:** 1 Department of Biology, University of Central Oklahoma, Edmond, Oklahoma, United States of America; 2 School of Biological Sciences, University of Sydney, Sydney, New South Wales, Australia; University of Utah, United States of America

## Abstract

Theory predicts the evolution of alternative male social tactics when intense competition coupled with the superior competitive ability of some individuals limits access to reproductive opportunities by others. How selection has shaped alternative social tactics may be especially interesting in long-lived species where size among sexually mature males varies markedly. We conducted experimental studies on long-lived eastern Australian water dragons living where competition was intense to test the hypotheses that mature males adopt alternative social tactics that are plastic, and that large size and body condition determine resource-holding potential. Approximately one-half of mature males (*N* = 14) defended territories using high rates of patrol and advertisement display, whereas 16 smaller mature males having lower body condition indices utilized non-territorial social tactics. Although territorial males were larger in absolute size and head dimensions, their heads were not allometrically larger. Territorial males advertised very frequently using displays involving stereotypical movements of the head and dewlap. More aggressive displays were given infrequently during baseline social conditions, but increased during periods of social instability. Female home ranges overlapped those of several territorial and non-territorial males, but females interacted more frequently with territorial males. The extreme plasticity of social tactics in this species that are dependent on body size was confirmed by two instances when relatively large non-territorial males spontaneously evicted territory owners, and by marked shifts in tactics by non-territorial males in response to temporary experimental removals of territory owners, followed (usually) by their expulsion when original owners were reinstated. The high level of social plasticity in this population where same-sex competitors are densely concentrated in preferred habitat suggests that chronic high energetic costs of defense may select for males to cycle between territorial and non-territorial social tactics depending upon their changing energetic status and their current capacity for competition with rivals.

## Introduction

Male vertebrates under strong sexual selection sometimes evolve alternative reproductive tactics (ART) characterized by distinctive behavior patterns, and/or morphological differences that are associated with resource holding potential ( = RHP). Alternative male tactics may be either genetically fixed, or ontogenetically plastic. When alternative tactics are fixed, males utilize one tactic throughout their lives [1]–[3]. In other cases, males switch between/among two or more alternative tactics [4], [5]. The usual case is for males to adopt socially dominant behavioral tactics, often defense of reproductive territories, when they obtain high RHP. High male RHP has been associated with large overall body size [6]–[9], allometric enlargement of particular body structures [9]–[11], conspicuous coloration [12], [13], secretion of certain chemical signals [14], and other traits that enhance performance in contests [15]. Males that are not able to acquire and defend territories may adopt subordinate tactics characterized by sneaking copulations when they can do so without being detected by socially-dominant males [16], [17], or by mimicking females [18]. Ascension to dominant social status by subordinate males often occurs through passive inheritance when socially dominant individuals die [5], [19]. However, especially in long-lived philopatric species, subordinate males may acquire territories by aggressive eviction of current territory owners [20], [21].

Diurnal lizards are among the best studied vertebrate examples of both fixed and conditional alternative male social tactics [4], [5], [18], [22]. Most research has involved lizard clades occurring in the New World and Europe, even though agamid lizards are conspicuous, diurnally active members of many habitats throughout Australia, Africa, and Asia. Moreover, some agamids are long lived, which likely increases the potential for the evolution of alternative tactics. Regardless of taxon, relatively few studies of lizards characterized by alternative male tactics have used experimental manipulations in the field to examine the mechanisms by which males switch from subordinate to dominant social tactics.

The eastern water dragon (*Physignathus lesueurii*), is common and conspicuous in riparian habitats throughout eastern Australia. Nonetheless, only two studies have addressed this species’ social ecology despite several observations suggesting that sexual selection may play a role in its social structure. Water dragons occur at high density along freshwater shorelines and individuals remain in relatively small areas [23]. This species shows male-biased sexual dimorphism in body size, head dimensions, and ventral coloration; red in mature males, brown/tan in females [24]. Moreover, because *P. lesueurii* is relatively large (up to 1 kg), it is probably long-lived [23]. Long-lived male iguanids typically display alternative reproductive tactics because individuals become reproductively mature before they acquire sufficient RHP to acquire and maintain dominant social status [18], [25], [26]. The large size and long life span of water dragons coupled with previous studies showing male-biased sexual dimorphism suggest strong potential for intense competition among males, and perhaps the evolution of alternative social tactics. We conducted experimental field studies to test two predictions related to male-male competition: 1) male water dragons will display alternative social tactics, and 2) social tactics will be conditional depending on resource holding potential related to overall body-size/dimensions and body condition. We report evidence to support both hypotheses. Because we documented spontaneous aggressive takeover of territories during our study (see below) and we prompted these experimentally, we also describe the behavioral dynamics of aggression during this process.

## Methods

### Ethics Statement

This research was conducted with the permission of the New South Wales National Parks and Wildlife Service (permit # S12905) and the Animal Ethics Committee, University of Sydney (L04/9–2009/1/5063).

### Study Population and General Methods

*Physignathus lesueurii* is a large (up to 1 kg, 304 mm snout-to-vent length = SVL) semiaquatic, diurnal agamid that occurs in riparian habitat throughout eastern New South Wales and southern Queensland [23], [27]. Adults are at least four years of age [23]. Water dragons are strong swimmers, and enter the water routinely to escape predators, and at night [28]. They feed primarily on terrestrial insects [29], but we also observed them eating aquatic crustacean and small lizards (Scincidae).

We conducted this study on the grounds of the Flynn’s Beach Resort in Port Macquarie, NSW (31° 26′ S latitude, 152° 55′ E longitude) from 12 Sept –30 Nov, 2009, during the Austral spring when eastern water dragons are reproductively active [23], [24], [29]. Water dragons occur throughout the resort grounds (8980 m^2^), but are highly concentrated along 0.2 km of Wright’s Creek which bisects this site. Microhabitat used by water dragons consisted of naturally vegetated riparian areas as well as lawns, plant beds, sidewalks, and even tiled swimming pool decks. Here, lizards were habituated enough to humans that they tolerated approach to within 2 m, but retreated when approached more closely. We captured 111 adult lizards by noose from 15–30 Sept, 2009, and then recaptured some of them once or twice. This number of lizards per 0.2 km of creek length extrapolates to an even higher estimate of density (555 lizards/km) than previously reported (138–215 lizards/km of shoreline) [23]. Lizards were marked by painting numbers on each side of the dorsal torso using white water proof nail polish. Worn numbers were retouched during recaptures, but none of the lizards lost their numbers through molting.

We determined the sex of individuals by 1) everting hemipenes, 2) palpating the abdomen for enlarging ovarian follicles, 3) inspecting head dimensions (larger in males), and 4) recording ventral coloration (more red-orange, red-black in males) [24]. We marked 36 females (SVL = 146–223 mm) in which we palpated enlarging ovarian follicles. We marked 38 males (SVL = 200–271 mm) with hemipenes that were secreting seminal fluid. Eight of these males were not observed on the study site after marking, but three of them moved a short distance onto property adjacent to the resort grounds. The remaining 30 males (SVL = 238–271 mm) were present throughout the study. We also marked 12 lizards (SVL = 192–214 mm) that lacked enlarging follicles, or eversible hemipenes, and had no more than 10 ventral scales that were light orange. We classified these lizards as immature, although they could have been mature females that did not produce eggs during the 2009 season [23]. There were also numerous (100–200) smaller lizards (60–160 mm SVL) throughout that we did not capture or mark. Previous estimates [23] indicate that water dragons of this size are juveniles up to 4 years old. Therefore, the larger, reproductively active adults that we focused on were considerably older.

### Social and Spatial Behavior

We mapped the study site to scale by recording distance and compass measurements among prominent, permanent landmarks (sidewalks, fence and light posts, trees, creek shore-line). The location of each mapping marker was determined using measurements among a minimum of five adjacent markers to yield a composite map accurate to the nearest 2 m. To determine use of space by mature females and males, we recorded at least one census of the entire study site each day from 24 Sept–26 Nov (*N* = 58 total censuses), except for 10 d when inclement weather prevented lizard activity. Censuses involved walking a routine path through the entire site and recording the location of all marked adults on maps. Daily census sightings were combined with the beginning and ending points of focal observations (described below) to determine the home ranges of individual lizards using the minimum convex polygon method [30]–[32]. The mean number of sightings used to map home ranges/territories was 28.8 (±2.0) for females, 87.1 for territorial males (±3.2), and 32.9 (±3.6) for non-territorial males (social status defined below). We used a planimeter (Planix 2000) to measure the areas and spatial overlap of home ranges/territories, and the linear interface of these with Wright’s Creek.

One of us (TAB) also recorded the behavior of lizards during focal observations [33]. Focal observations involved recording all of the displays and aggressive encounters with conspecifics of both sexes initiated by subject lizards [16]. Although lizards at this site were not overtly affected by human presence, we recorded focal observations when human disturbance was minimal. Each observation session lasted 20 min, except for a few (<5%) that were ended 1–5 min earlier because focal lizards were lost from view under thick vegetation. We recorded a minimum of five focal observations during baseline social conditions on separate days on 30 mature males, and one focal observation on each of 17 mature females. We used principal components analysis (PCA) with Variamax rotation of rate of patrol (m/h), total displays/min, aggressive encounters initiated with rival males/min, and percent intrasexual encounters won or tied to further examine behavioral variation among these 30 mature males.

### Male Morphometry

We recorded nine morphometrics that have been associated with success and performance in agonistic contests in other lizard species [6], [34], [35]. These were: SVL - the tip of the snout to the vent along the ventral midline, tail-length – posterior tip of the tail to the vent (both ±1 mm), total body mass (±5 g), three head dimensions ±0.1 mm (width - at the jaw articulation where it is widest, length - from the tip of the upper jaw to the anterior edge of the tympanum, depth - from the top to the bottom behind the eyes where greatest with the jaws held closed), length of the hind- and front legs - both on the right side from the anterior insertion on the torso to the distal end of the longest digit when the limb was held at a right angle, and the cumulative length of the three longest dorsal crests on the head. We also recorded the number of scars. The residuals of a regression of log SVL on log total body mass (*F*_1,29_ = 22.08, *P*<0.0001, *r* ^2^ = 0.44) was used as an index of body condition.

We first summarized variation among mature males in their absolute size and body dimensions by calculating principal components analysis on nine measurements not corrected for body size. Because behavioral data indicated that mature males adopted two distinct social tactics (territorial and non-territorial; see Results below), we used non-paired *t*-tests to compare morphometric factor scores on the nine non-size-corrected measurements of males in these two social categories. All body dimensions were positively correlated with SVL (*r*- values = 0.382–0.864, *P* values = 0.037−<0.0001) except tail length (*r* = 0.012, *P* = 0.948). Therefore, to examine the possibility that territorial and non-territorial males showed allometric variation, we corrected for variation in body size by regressing eight log-transformed body dimensions on log-transformed SVL and calculating the residuals [35]. We then calculated PCA on the residuals of these eight variables, and compared the factor scores for the size-corrected measurements of territorial and non-territorial males.

### Spontaneous Territory Takeovers and Removal Experiments

Before we could perform removal experiments (see below) to test whether alternative behavioral tactics displayed by water dragon males are fixed or plastic, we observed two spontaneous takeovers of territories where a previously non-territorial male challenged, defeated, and evicted a male territory owner for which we had previously recorded baseline observations for both males, and recorded observations during and following the eviction. We then partially mimicked these instances by conducting 11 trials where we removed a territorial male (a different individual in each trial) at 1400–1600 h and kept him off site for 2 d. Removed males were maintained in damp, low light conditions to mimic cool, cloudy, rainy field conditions when water dragons are inactive. From 0730–1500 h during the 2 d removal, we recorded at least three, 20-min focal observations on all of the mature males adjacent to the territory of the removed male. After nightfall on the second removal day (2000–2100 h), we returned the removed male to his original territory. On the following day (0900–1500 h) we recorded at least three, 20-min focal observations on the males that had been present during the removal, plus focal observations on the reinstated territorial male. We used repeated-measures analysis of variance (ANOVA) to test for behavioral differences during the three phases (pre-removal, during removal, reinstatement) of our removal experiments.

## Results

### Male Social Behavior and Alternative Tactics

During baseline social conditions, 98% of displays given by males were stereotypical movements of the head alone. Head raises involved lifting the head abruptly to a 45° angle, which was almost always followed by bobbing it ( = head bobs) vertically as many as eleven times in succession [24]. Much less often, males raised the head gradually at first but then abruptly jerked it upward to its highest angle. Dorsal crests on the head were usually erected during head displays. Males also occasionally displayed by rolling their tails vertically from the tip to base in a smooth motion, or by lateral movements of the slightly raised tail.

More aggressive displays were infrequent during baseline conditions, but became more common during escalated social encounters that characterized unstable social conditions (described below). Males sometimes began escalated encounters by charging an opponent while running on only the hind legs and flailing their front limbs (bipedal charges). More usually, encounters began with “full shows” which involved tilting the head upward at a 30–40° angle while holding the mouth open partially, extending the dewlap, slowly elevating on all four legs and compressing the torso laterally and then lowering. Males sometimes performed only one full show which they held for a few seconds before lowering, but other times they elevated and lowered as many as four times in succession. When rival males approached more closely (≤5 body lengths), opponent males elevated and laterally compressed their torso while running sideways toward their rivals (3–15 steps) using a stiffened gait and with the dewlap extended ( = display runs).

Under baseline social conditions, 14 of the mature males were sighted on all (100%) censuses, which was statistically more frequent (*F = *_2,62_ = 113.6, *P*<0.0001) than the mean (*x* = 56.9%, SE = 3.6) for the other 16 mature males. A significant (*P*<0.0001) PCA on the frequency of social behavior patterns revealed two factors that together explained 83.7% of the total variance in male behavior. Rate of patrol, total displays/minute, and percent encounters won or tied loaded together on Factor 1 (58.8% of total variance), whereas only intrasexual encounters initiated/min loaded on Factor 2 (24.9% of variance) (Table 1). The mean score for Factor 1 was higher (*t*_1,28_ = 8.29, *P*<0.0001) for the 14 males that were sighted on all censuses, whereas the means for Factor 2 (frequency of intrasexual aggression) were not different (*t*_1,28_ = 0.79, *P* = 0.438) in these two groups of males. Based upon these marked differences in Factor 1 scores and differences in sighting frequencies on censuses, both of which we interpret as indications of the extent to which males were advertising ownership to rivals, we classified males as territorial (*N* = 14) and non-territorial (*N* = 16) for subsequent analysis.

**Table 1:** Principal component factor loadings for social behavioral variables during baseline conditions.

Behavioral Variable	Factor 1(58.8%)	Factor 2(24.9%)
Patrol (m/h)	**0.810**	0.310
Total displays/min	**0.891**	−0.007
Aggresssive encounters Initiated/min	−0.269	**0.949**
% contests won or tied	**0.912**	0.120

Parenthetical numbers are the percent of total variance explained by each factor. High loading factors are in boldface.

Comparison of individual behavioral variables revealed that average rate of patrol by territorial males was twice (*U*_1,28_ = 183, *P* = 0.0003) that of non-territorial males (Table 2). Rates of head-raise and head bob displays, were each 6.7 times those of non-territorial males (*U*_1,28_ - values_ = _216.0, 210.5, respectively, both *P*–values <0.0001), and the frequency of all displays pooled by territorial males was also higher (*U*_1,28_ = 210.5, *P*<0.0001). Territorial males usually either repelled non-territorial rivals, or tied in contests with neighboring territory owners on shared borders (Table 2). By contrast, non-territorial males lost almost all encounters with territorial males (except during spontaneous takeovers described below), but prevailed in encounters with smaller juvenile males.

**Table 2:** Behavior of male water dragons during baseline social conditions.

Behavioral Variable	Non-Territorial (*N = *16)		Territorial (*N = *14)
Patrol (m/h)	13.3 (1.8)	*	26.4 (3.2)
Aggressive encounters/min	0.021 (0.004)		0.033 (0.009)
Head-up displays/min	0.03 (0.01)	*	0.18 (0.02)
Head-bob displays/min	0.13 (0.04)	*	0.81 (0.10)
Total displays/min	0.16 (0.05)	*	1.01 (0.46)
% wins + draws	7.2 (2.9)	*	90.4 (4.9)

Data are means ±1.0 standard error. Asterisks indicate statistically significant differences (*P* – values = 0.05–0.0001) between territorial and non-territorial males.

We anecdotally witnessed only five instances of short chases between females. However, neither aggression nor advertisement displays by females were observed during focal observations, even though they routinely basked within 5 m of at least one consexual.

### Male Morphometry

A significant (*P*<0.0001) PCA on nine non-size-corrected measurements revealed three factors that together explained 80.7% of the morphometric variation among mature males (Table 3). SVL, all three head dimensions, front leg length, body mass, and the cumulative length of the three longest dorsal crests all loaded highest on Factor 1, which explained 54.9% of the total variance. Hind leg length and tail length each loaded highest on Factors 2 and 3 (Table 3) and explained 13.7 and 12.2% of the total variance, respectively. The mean score for Factor 1 was higher (*t*_1,29_ = 2.29, *P* = 0.030) in territorial males (*x* = 0.42, SE = 0.15) than non-territorial males (*x* = −0.37, SE = 0.29), whereas the two groups of males had similar mean scores for Factor 2 (*t*_1,29_ = 0.47, *P* = 0.65) and Factor 3 (*t*_1,29_ = 1.68, *P* = 0.104). Estimates of body condition were higher (*t*_1,29_ = 2.50, *P* = 0.019) in territorial (*x* residual score ±1.0 SE = 0.46±0.29) than non-territorial males (−0.42±0.21).

**Table 3:** Principal component factor loadings for male water dragon morphometric variables.

	Non Size-Corrected			Size-Corrected		
Morphometric Variable	Factor 1(54.9%)	Factor 2(13.7%)	Factor 3(12.2%)	Factor 1(34.3%)	Factor 2(17.2%)	Factor 3(14.8%)
Snout-to-vent length	**0.915**	0.095	0.087	–	–	–
Body mass	**0.744**	−0.442	−0.067	**0.603**	−0.455	0.066
Tail length	−0.005	−0.495	**0.829**	−0.038	−0.533	**0.600**
Head width	**0.891**	−0.136	−0.016	**0.794**	−0.006	0.052
Head length	**0.936**	0.041	−0.054	**0.727**	0.356	0.036
Head height	**0.801**	−0.276	−0.080	**0.801**	−0.098	0.239
Cumulative dorsal crest length	**0.792**	0.114	−0.340	**0.717**	0.475	−0.064
Hind leg length	0.350	**0.783**	0.384	−0.347	**0.696**	0.338
Front leg length	**0.696**	0.256	0.343	−0.127	0.196	**0.799**

Parenthetical values are the percent variance explained by each factor. High loading factor scores are in boldface.

A significant (*P*<0.008) PCA analysis on eight size-corrected measurements also revealed three factors that together explained 66.4% of the total morphometric variation among males. All three head dimensions, the cumulative length of the three longest dorsal crests loaded highest on Factor 1 which explained 34.3% of the total variance (Table 3). Hind leg length alone loaded highest on Factor 2 explaining 17.2% of variance, and tail length and front leg length loaded on Factor 3 which explained 14.8% of the variance (Table 3). There were no significant differences in any of the factor scores (*t*’s = 0.52–1.29, *P*’s = 0.21–0.61) between territorial versus non-territorial males.

### Use of Space and Intersexual Interactions

Areas occupied by all mature males included some shoreline of Wright’s Creek, but the relative amount of shoreline was higher (*F*_2,62_ = 8.26, *P* = 0.0007) in non-territorial males than both territorial males and females (Table 4). Male social tactics and sex influenced the amount of area used by water dragons (*F*_2,62_ = 16.5, *P*<0.0001). Non-territorial males used 1.7 and 4.2 times more space than territorial males (*P* = 0.015) and females respectively (*P*<0.0001; Table 4). Male territories were larger (*P = *0.014) than female home ranges. Home ranges of females partially overlapped with as many as eight other female home ranges, resulting in very little home range area exclusive of other females (Table 4). Territorial males had 2–6 territorial neighbors, but they controlled exclusive use of more than 75% of their territory from these neighboring rivals. By contrast, portions of the home ranges used by non-territorial males were overlapped by 2–13 other non-territorial males as well as 2–7 territorial males, such that none of the area used was exclusive of space used by same-sex rivals (Table 4).

**Table 4:** Use of space by territorial and non-territorial male, and female water dragons.

Spatial Variable	Non-Territorial Males (*N = *16)		Territorial Males (*N* = 14)		Females (*N = *36)
Area (m^2^)	731.9 (154.1)	*	434.9 (40.9)+	*	174.2 (18.8)
% area exclusive from consexuals	0(0.0)		78.5 (5.1)		15.2 (3.8)
Ratio of home range shoreline to perimeter	0.48 (0.49)++	*	0.34 (0.46)	*	0.31 (0.22)

Data are means ±1.0 SE. Asterisks indicate statistically significant differences (*P*<0.05) between adjacent columns. The single plus sign indicates a significant difference between territorial males and females, double plus sign indicates a statistical difference between non-territorial males and females.

**Table 5:** Responses by non-territorial water dragon males to temporary removal and reinstatement of individual territorial males.

Variable	Pre-Removal		During Removal		Reinstatement
Displays/min +	0.27 (0.07)	*	1.81 (0.14)	*	0.52 (0.17)
Patrol (m/h)	15.5 (3.4)	*	61.8 (7.5)	*	29.7 (9.3)
% wins + draws +	17.8 (7.2)	*	79.4 (9.3)	*	16.7 (11.2)

Data are means ±1.0 standard errors. Plus signs indicate statistically significant effects (Repeated measures ANOVA, *P*<0.001) of experimental phase. Asterisks indicate statistically significant differences (*P*<0.05) between adjacent columns.

Home ranges of 17 (of 36) females were overlapped by one territorial male, whereas nine, seven, and three females were overlapped by two, three, and four territorial males respectively. Similarly, 2–8 non-territorial males partially overlapped the home ranges of all 36 females. The cumulative result was that at least 90% of all of the area occupied by females was overlapped by several mature males. Despite extensive spatial overlap by both classes of males (see below), females interacted much more frequently (*t*_1,28_ = 4.18, *P* = 0.0003) with territorial males (*x* encounters/hour ±1.0 SE = 1.15±0.13) than non-territorial males (0.32±0.15). The most common type (98%) of interaction between the sexes involved males approaching a female to within one body length and the female lowering her head, arching both her back and proximal tail dorsally while holding the distal end of the tail stiffly on the ground. Females gave this display either when they remained in place or when walking slowly forward as the male approached. Males usually only made brief contact with displaying females and then moved past them. During focal observations, we observed one attempted copulation and three instances when a male and female sat for several minutes with one lizard partially superimposed over the other. During censuses, we observed 22 other instances of intersexual physical contact, and all of these involved territorial males.

### Spontaneous Takeover of Territories

We recorded two spontaneous takeovers of territories by males that were not territorial at the beginning of our study. In both instances, the non-territorial males engaged a territory owner and defeated him during one highly aggressive encounter involving prolonged (up to 1 h) full shows, display runs, bipedal chases (described above), and especially, intensely physical fights characterized by bites that produced injury (described below). As a consequence of these single decisive fights, winners increased rates of patrol, and head displays 1.0–2.8– fold respectively, whereas these behavior patterns decreased 2.3–4.3-fold in the losers. In both instances, the non-territorial males that took-over territories were larger in snout-to-vent length and mass, and both males held their usurped territory for the remainder of the study (35 and 51 d). The expelled males remained in these same areas, but adopted socially subordinate behavior at least until the end of our study.

### Male Removal Experiments

In 10 of 11 removal trials, one overlapping non-territorial male exhibited marked shifts in social tactics. Two non-territorial males responded similarly in the other trial. These initial responses began within 2 h of first sunlight on the first removal day. Responses involved marked increases (Table 5) in rates of display (repeated-measures ANOVA, *F*_1,11_ = 31.80, *P*<0.001), and patrol (*F*_1,11_ = 12.85, *P* = 0.0002). The single respondent in ten trials quickly prevailed using mostly displays and bipedal charges in encounters with other non-territorial males, and increased (*F*_1,11_ = 14.21, *P*<0.0001) the frequency of encounters that they won or fought to a tie to establish occupancy of the territory by 1200 h of the first removal day (Table 5). In the other trial, two non-territorial males (snout-to-vent lengths = 237 and 256 mm) fought intermittently from 1330–1530 h of the first removal day before the larger male prevailed and the smaller male withdrew. On the second removal day, all of the newly established males continued their higher rates of display and patrol, and winning or at least fighting to a draw with all rivals.

Nine different non-territorial males responded during the 11 removal experiments. Two of these were animals that had very large home ranges that partially overlapped the territories of several males, and each responded in two and three removal trials respectively. They contested one another in the trial where two males responded (described above). Seven different non-territorial males responded in the other trials. There were nine additional mature non-territorial males that also overlapped the territories of removed males, but these immediately fled in all 18 interactions with respondent males, and their very low rates of prior display (*x* displays/min ±1.0 SE = 0.09±0.02), did not change (*t*_1,14_ = 0.92, *P* = 0.38) during removals (*x* = 0.12±0.04). These non-responding, non-territorial males were smaller in SVL (*t*_1,14_ = 2.36, *P* = 0.03), total mass (*t*_1,14_ = 4.43, *P* = 0.004), all three head dimensions (*t’s*_1,14_ = 3.09–3.86, *P’s* = 0.001–0.007), and had lower body condition indices (*t*_1,14_ = 5.05, *P* = 0.001), than responding non-territorial males. They did not differ significantly (*t’s*_1,14_ = 0.13–1.51, *P’s* = 0.15–0.9) in mean tail length, length of the front- and hind-limbs, and the cumulative length of the longest three dorsal crests.

**Figure 1:**
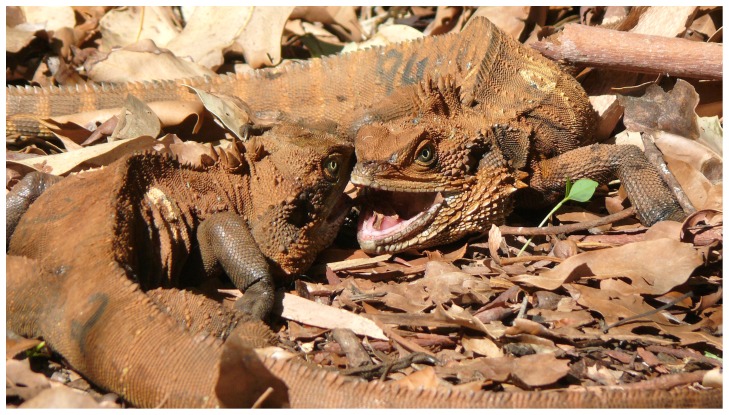
Male water dragons engaged in a prolonged escalated contest.

Reinstatement of original territory owners elicited the highest intensity contests that we observed. Aggression began as soon as original owners first confronted new owners when removal sites became sunlit (0730–0800 h). Contests began with males approaching opponents giving head displays, full shows, display runs, and/or bipedal charges (described above). Aggression escalated with males confronting one another, snout-to-snout within one head length, and then butting the sides of their heads against that of their rival. In this position, each male pushed forcefully in opposite directions using the torso, limbs, and tail to gain leverage and traction (Figure 1). Both males were sometimes able to bite and clamp on their rival’s head or front limbs, and/or forcefully twist their opponent and even hold him underwater when fights occurred along the shoreline. Fighting males separated intermittently (one body length or less) during which they breathed rapidly and deeply before re-engaging. Most such contests lasted 30–40 min, but the fight between two rival non-territorial males on day 1 of one trial (described above) continued intermittently for 2 h, and the most intense contest that ensued following reinstatement of an original owner continued uninterrupted for 160 min. Although these intense fights were the consequence of removal/reinstatement experiments, a higher number of scars (*F*_1,62_ = 19.04, *P*<0.0001) in both territorial (*x* number of scars ±1.0 SE = 6.8±1.2) and non-territorial males (*x* = 4.7±1.1) than females (*x* = 0.6±0.4) indicates that fights among males that produce injuries are common in this population.

Upon their reinstatement, in 9 of 11 trials the original territory owners defeated and repelled the males that changed to using territorial tactics, causing rates of patrol and display by male losers to decrease (*P* = 0.01 for both tests, Table 5). In two trials, the newly established owners defeated and repelled the original owners, and continued to occupy their territories for at least the remainder of the study (only 5 and 11 d because these trials were conducted toward the end of our study). In one of these instances, the removed male’s lower jaw was fractured at the mandibular symphysis and the surrounding soft tissue was infected. This male did not vigorously contest the new owner when we reinstated him. In the other trial, the contesting males were identical in SVL (both 248 mm) but the newly victorious male was 10 g heavier. The contest that ensued during the reinstatement phase of this trial was the longest (160 min) bout of aggression that we observed.

## Discussion

Theory addressing habitat settlement in territorial species often assumes that individuals gain territories by either inheriting them following mortality of previous owners, or by colonizing vacant areas [20], [36], [37]. Examples of mortality-induced territory inheritance are well documented in a variety of vertebrate taxa (e.g., birds [38]–[41], fish [19], lizards [5]). Territory acquisition by forcible eviction of current territory owners, like that observed twice during only a short study on water dragons, is much more rare but has also been documented in insects [42]–[45], fish [45], birds [20], [21], [46], mammals [47], and at least one lizard [48]. Together, our observations of spontaneous evictions of territory owners, rapid territory takeovers in response to experimental removals, high baseline rates of advertisement display by territory owners, as well as prominent scars on large males all indicate that intrasexual male competition is intense in this high density population of water dragons, which likely makes the costs of territory maintenance very high.

Marked variation in the baseline behavior of mature males clearly supports the hypothesis that sexually mature water dragon males utilize two social tactics (territory defense and non-territorial satellite behavior). Moreover, territory takeovers by aggression, both spontaneously and in response to removals, show that alternative male tactics are plastic. A significant role of absolute size in RHP is indicated by the larger snout-to-vent length and head dimensions of territory owners under baseline conditions as well as the outcome of contests provoked by removals in relation to size. There was no indication that the head dimensions of territory owners were allometrically larger than those of non-territorial males; resource-holding potential simply increases as males grow isometrically. Reacquisition of territories by original owners in most trials may also be promoted by prior residency advantages [49], [50]. Future analyses of the behavioral dynamics during the prolonged interactions that we recorded will evaluate the extent to which asymmetries in male size and prior residency may explain contest resolution in water dragon males in relation to the predictions of game-theory models (TA Baird, TD Baird, & R Shine unpublished data).

Although field studies on agamid social behavior are still sparse, there is growing evidence of the evolution of alternative male tactics in this clade. Male defense of territories has been reported in several species [51]–[54]. In at least two species where some males defend territories, alternative tactics are suggested by the observation that other individuals adopt subordinate behavior patterns when they are near to defended areas [52], [55]. Male-biased sexual dimorphism in body dimensions [56], [57] and coloration [58] are also consistent with a role for sexual selection in the social systems of still other agamids. Moreover, advertisement displays involving stereotypical head movements, dewlap extension, lateral compression and vertical elevation of the torso, as observed in water dragons and other agamids, are similar to those described for many new world sexually-selected iguanids [59]–[61], and suggest intense sexual selection and the evolution of male ART. Despite these general similarities, water dragons differ from other agamids in the relative frequency that they use various types of advertisement displays. The most frequent displays by male water dragons involved head movements, whereas tail displays (infrequent in *Physignathus*), are prominent in both jacky dragons [62], [63] and frillneck lizards [57]. Interspecific variation in display use may be a consequence of factors that influence signal transmission and reception in the particular habitats occupied by these species.

The primary benefit for males of defending territories during the breeding season is access to receptive females [64]. Consistent with this prediction, territorial male water dragons interacted more frequently with females that they overlapped than did non-territorial males. Nonetheless, social dominance and interactions do not necessarily equate with exclusive reproductive access in squamate reptiles, including agamid lizards [65]. For example, in a population of ornate dragons, in which both males and females defended territories, 25% of female clutches were fertilized by multiple males, 65% of clutches were partially sired by males other than the overlapping territorial male, and 35% of clutches were partially fertilized by males having no observed spatial overlap with females that they sired offspring with [53]. Given the extensive overlap of female water dragon home ranges by mature territorial and non-territorial males, the distribution of reproductive success among males of this species in relation to their social tactics remains an open question.

High chronic costs of territory defense in our population are indicated by the frequent (∼1 per min) advertisement displays, intense contests that produce injuries, and spontaneous territory takeovers. When costs of territory maintenance are chronically high, selection may favor mechanisms to reduce them [50], [66], [67]. One possibility is that high chronic defensive costs may select for the evolution of signals requiring a range of energetic expenditure and graded content (iguanians [16], [68]; chameleonids [69]; varanids [70]; other agamids [62], [63]. Consistent with this hypothesis, the most frequent displays by water dragon males involved movements of the head, which likely require only low expenditure of energy. More aggressive behavior patterns that almost certainly require higher energy expenditure were given only infrequently when social conditions were stable, but these increased when social encounters intensified into highly aggressive disputes over territory ownership.

The high density of water dragon males in relation to preferred habitat (creek shoreline) appears to result in chronic intense competition that probably exacts a high cumulative energetic cost. On our site, competition among sexually-mature males was intense enough to prevent several large individuals from controlling territories having preferred shoreline. Instead, these males adopted subordinate satellite tactics to remain within this high-quality habitat, but remained poised to compete aggressively for territories when opportunities arose. Territory acquisition tactics included spontaneous aggressive challenges which were sometimes successful in evicting territory owners, perhaps because these territorial males had been weakened by the high chronic costs of advertisement and defense imposed by the intense level of chronic competition in this population. Because water dragon males are long-lived, and both large size and high body condition are correlated with their social status, the chronic high energetic demands necessary to both sustain peak physical condition and advertise/defend territory occupancy may require males to cycle between aggressive (territorial) and less aggressive (non-territorial) social tactics to manage their life-time energetic costs. The most similar situation reported for other lizards appears to be the density-induced shifts among three conditional social tactics in male marine iguanas [18], which are also long lived and occupy habitats where high-quality territories are limited [71], [72]. Usurpation of territories by evicting owners and cycling between alternative social tactics also occurs in dense populations of long-lived birds. For example, loons engage in intense, costly territorial contests, and eviction of territory owners is common [21]. Territory acquisition by eviction is also common in song-sparrows that survive several seasons and live at high density on islands from which they do not migrate [20]. Hence, shifting between low aggression - low cost, and more aggressive - higher cost social tactics may be an optimal strategy for long-lived sedentary males where the demands of defending territories is chronically high owing to the ever present large number of intrasexual competitors.
